# Clinicopathological Criteria Predictive of Recurrence Following Bacillus Calmette-Guérin Therapy Initiation in Non–Muscle-Invasive Bladder Cancer: Retrospective Cohort Study

**DOI:** 10.2196/25800

**Published:** 2021-06-22

**Authors:** Joseph Plasek, John Weissert, Tracy Downs, Kyle Richards, Kourosh Ravvaz

**Affiliations:** 1 Advocate Aurora Research Institute Milwaukee, WI United States; 2 School of Medicine and Public Health University of Wisconsin-Madison Madison, WI United States

**Keywords:** urinary bladder neoplasms, risk factor, bacillus Calmette-Guérin, recurrence

## Abstract

**Background:**

Bacillus Calmette-Guérin (BCG) is currently the most clinically effective intravesical treatment for non–muscle-invasive bladder cancer (NMIBC), particularly for patients with high-risk NMIBC such as those with carcinoma in situ. BCG treatments could be optimized to improve patient safety and conserve supply by predicting BCG efficacy based on tumor characteristics or clinicopathological criteria.

**Objective:**

The aim of this study is to assess the ability of specific clinicopathological criteria to predict tumor recurrence in patients with NMIBC who received BCG therapy along various treatment timelines.

**Methods:**

A total of 1331 patients (stage Ta, T1, or carcinoma in situ) who underwent transurethral resection of a bladder tumor between 2006 and 2017 were included. Univariate analysis, including laboratory tests (eg, complete blood panels, creatinine levels, and hemoglobin A_1c_ levels) within 180 days of BCG therapy initiation, medications, and clinical and demographic variables to assess their ability to predict NMIBC recurrence, was completed. This was followed by multivariate regression that included the elements of the Club Urológico Español de Tratamiento Oncológico (CUETO) scoring model and variables that were significant predictors of recurrence in univariate analysis.

**Results:**

BCG was administered to 183 patients classified as intermediate or high risk, and 76 (41.5%) experienced disease recurrence. An abnormal neutrophil-to-lymphocyte ratio measured within 180 days of induction BCG therapy was a significant predictor (*P=.*047) of future cancer recurrence and was a stronger predictor than the CUETO score or the individual variables included in the CUETO scoring model through multivariate analysis.

**Conclusions:**

An abnormal neutrophil-to-lymphocyte ratio within 180 days of BCG therapy initiation is predictive of recurrence and could be suggestive of additional or alternative interventions.

## Introduction

### Background and Significance

Bacillus Calmette-Guérin (BCG) is currently the most clinically effective intravesical treatment for non–muscle-invasive bladder cancer (NMIBC), particularly for patients with high-risk NMIBC such as those with carcinoma in situ (CIS). Unfortunately, recent manufacturing insufficiencies have resulted in a worldwide, health-threatening BCG shortage [1-4]. Optimizing the limited supply by identifying patients who could benefit the most from BCG treatment is essential from a public health perspective because the percentage of patients with NMIBC who fail BCG treatment has been reported to be as high as 40% [5]. BCG optimization could also improve patient safety by reducing BCG treatments that have a low probability of improving clinical outcomes [6]. The incidence of BCG-related adverse effects is considerable—nearly 70% of the patients with NMIBC in a large, randomized controlled trial experienced local or systemic complications, including a long-term risk of treatment sequelae that can develop years after BCG therapy initiation [7,8]. In an era of BCG shortage, a prediction model that could predict NMIBC recurrence following BCG therapy initiation (ie, BCG failure) could support both public health and precision medicine. A risk-adapted approach for BCG maintenance therapy could continue to minimize treatment-related toxicity and optimize cost-effectiveness regardless of BCG availability in the future [4].

The heterogeneous risk of cancer recurrence and progression in patients with NMIBC has led to the investigation of a wide range of methods and factors for predicting a patient’s prognosis, including the Club Urológico Español de Tratamiento Oncológico (CUETO) scoring model, which was designed for patients treated with BCG, and European Organization for Research and Treatment of Cancer nomograms [9]. Although the CUETO scoring model and European Organization for Research and Treatment of Cancer nomograms are the most widely used predictive models to date, their accuracy is inconsistent, and the search continues to find better clinical, pathological, genetic, or demographic prognostic features, alone or in combination [10-12]. In studies with patients who received BCG treatment, the findings suggest that recurrence and disease progression may be predicted by indicators of health (eg, BMI) and measures of inflammation (eg, an elevated neutrophil-to-lymphocyte ratio) [13-16]. Other novel prognostic measures for BCG recipients include immunological or cytokine-based markers (eg, urinary fluorescence in situ hybridization testing and urinary cytokine-based nomograms), protein-based biomarkers (eg, ezrin), and gene-based biomarkers (eg, quantifying mutations in DNA damage repair genes) [8].

### Objective

Debate and uncertainty persist regarding the potential of various clinical risk factors. The overarching goal of this study is to identify and validate easily employable risk factors that predict BCG failure. Although immunological or cytokine-based markers, protein-based biomarkers, and gene-based biomarkers show potential, they require extra expenditure and testing because they are not collected in the normal course of clinical care. In contrast, clinicopathological criteria are often captured as part of the clinical workflow and are thus actionable at the time that treatment decisions are being made. The aim of this study, therefore, is to assess the ability of commonly used clinicopathological criteria or medications to predict recurrence in patients with NMIBC who were treated with intravesical BCG.

## Methods

### Recruitment

We conducted a retrospective cohort analysis following institutional review board (IRB) approval. From 2006 to 2017, a total of 1331 patients underwent transurethral resection (TUR) of a bladder tumor that was clinically staged as non–muscle invasive. Patients received care within a community health care system in the Midwest that includes 17 different hospitals dispersed across a wide geographic area that spans rural, suburban, and urban locations. Data captured during the normal course of clinical care were extracted and collated retrospectively from the cancer registry (ie, patient demographics, cancer diagnosis, recurrence, and treatment) and electronic health records (EHRs; ie, surgery and pathology reports, medication orders, laboratory tests, procedure codes, and billing diagnoses). We used a hybrid data extraction and preparation pipeline incorporating automated, semiautomated, and manual techniques. A detailed description of the pipeline can be found in our previous study [17]. This study was performed in compliance with the World Medical Association Declaration of Helsinki on Ethical Principles for Medical Research Involving Human Subjects and was reviewed and approved by the IRB. The IRB waived the requirement for informed consent because of the low risks of the study.

We included patients with NMIBC and a primary or recurrent diagnosis of Ta or T1 urothelial carcinoma or CIS per the American Joint Committee on Cancer tumor size, node involvement, and metastasis system [18]. Patients were excluded from the study if they had metastatic urothelial carcinoma. Patient follow-up continued from the initial TUR (index TUR) until recurrence, progression (ie, ≥stage T2), cystectomy, death, or last known bladder cancer–directed treatment (ie, cystourethroscopy, TUR, urologist visit, BCG instillation, chemotherapy instillation, or urine cytology test). Only patients newly diagnosed with NMIBC on the index TUR were enrolled into this study.

### Measures

Recurrence following the index TUR was the primary outcome of interest in this study. Recurrence was defined as cancer returning more than 6 weeks after the index TUR. In contrast, a TUR occurring 2-6 weeks after the index TUR was defined as a Re-TUR (ie, a second-look TUR) instead of a recurrence. Progression, in this study, was defined as cancer upstaging to ≥stage T2 or patients requiring cystectomy. Bladder cancer recurrence and progression were identified using pathology reports and the dates of the events listed in the cancer registry. The date of decease was also captured in the cancer registry. The BCG instillation date was extracted from (1) procedure billing data, (2) medication administration records, (3) medication order records (ie, prescription), or (4) extracted from a free-text chemotherapy field in the cancer registry data.

Pathology reports were reviewed to determine tumor stage, size, quantity, and grade for each TUR. The tumor stage recorded was the highest stage confirmed by the pathologist. Tumor grade was captured in, or converted to, the 2004 World Health Organization grading system. Tumor size was stratified into small (≤3 cm) or large (>3 cm). CIS and lymphovascular invasion were extracted from the cancer registry. The study cohort is exclusively composed of patients with a primary cancer diagnosis; therefore, all patients with a prior incidence of NMIBC were excluded. Variant histology was extracted from the pathology reports, although all patients with variant histology in the data were staged T2 or higher in their index TUR and, thus, were excluded from the study. High-grade prostatic urethral involvement occurs when the cancer preferentially invades the prostatic urethra before the bladder muscle and was extracted from the pathology reports. Age was calculated based on the difference between the date of the index TUR and date of birth and was extracted from the cancer registry (along with sex).

Several clinical characteristics associated with the efficacy of BCG treatment were extracted from the EHR, including white blood cell, lymphocyte, neutrophil, monocyte, and platelet counts and levels of creatinine and hemoglobin A_1c_ (Table 1). The derived neutrophil-to-lymphocyte ratio and platelet-to-lymphocyte ratio were computed based on the EHR data. We investigated three different time frames corresponding to pre-TUR, 180 days after TUR, and beyond 180 days after TUR (Multimedia Appendix 1). The pre-TUR timeframe corresponds to prior risks. A +1- to +180-day after TUR time frame covers the induction BCG and early maintenance BCG treatments. The delta in laboratory values was calculated to further evaluate whether a change in the patients’ baseline clinical characteristics following BCG treatment demonstrated any clinical relevance in predicting recurrence (Multimedia Appendix 1). Patients who received BCG did not have an estimated glomerular filtration rate or tuberculosis status noted for the purposes of this study.

**Table 1:** Clinicopathological criteria or medication definitions.

Type and clinicopathological criterion or medication		Description
**Binary**		
	BCG^a^ instillation	First instillation of BCG documented 0-90 days after TUR^b^
	Epirubicin	Epirubicin use documented 0-90 days after TUR
	Tuberculostatic agents	Use of isoniazid isonicotinylhydrazide, rifampicin, rifambutin, fluoroquinolones (ofloxacin, ciprofloxacin, levofloxacin, and moxifloxacin), ethambutol, clarithromycin, aminoglycosides (gentamicin, amikacin, tobramycin, kanamycin, and neomycin), or doxycycline documented –30 to 90 days of induction BCG (index to median day of BCG administration if no BCG)
	Spasmolytics or anticholinergics	Use of spasmolytics: oxybutynin documented –30 to 90 days of induction BCG (index to median day of BCG administration if no BCG)
	Antiphlogistics	Use of antiphlogistics: fluticasone documented –30 to 90 days of induction BCG (index to median day of BCG administration if no BCG)
	Topical steroids	Use of local topical steroids: betamethasone, clobetasol, diflorasone, fluocinoide, halobetasol, amcinonide, desoximetasone, propionate, triamcinolone, fluocinolone, hydrocortisone, desonide, alclometasone, and mometasone documented –30 to 90 days of induction BCG (index to median day of BCG administration if no BCG)
	Nonsteroidal anti-inflammatory drugs	Use of nonsteroidal anti-inflammatory drugs: aspirin, ibuprofen, naproxen, nabumetone, celecoxib, diclofenac, etodolac, indomethacin, ketoprofen, ketorolac, and piroxicam documented –30 to 90 days of induction BCG (index to median day of BCG administration if no BCG)
**Numeric**		
	General description	Most recent (laboratory test) occurring 1-180 days after induction BCG computed 14 days before the next event (ie, TUR but not Re-TUR^c^, recurrence, progression, or death) Difference between the most recent (laboratory test) occurring 1-180 days after induction BCG computed 14 days before next event and the most recent (laboratory test) occurring 90-0 days before induction BCG (index to median day of BCG administration if no BCG)
	Lymphocyte count (normal: 20%-40% differential)	N/A^d^
	Neutrophil count (normal: 55%-70% differential)	N/A
	Monocyte count (normal: 2%-8% differential)	N/A
	Platelet count (K/μL)	N/A
	White blood cell count (K/μL)	N/A
	Creatinine level (mg/dL)	N/A
	Hemoglobin A_1c_ (mmol/mol)	N/A
**Computed percentage**		
	General description	Most recent (laboratory test) occurring 1-180 days after induction BCG computed 14 days before the next event (ie, TUR but not Re-TUR, recurrence, progression, or death) Difference between the most recent (laboratory test) occurring 1-180 days after induction BCG computed 14 days before next event and the most recent (laboratory test) occurring 90-0 days before induction BCG (index to median day of BCG administration if no BCG)
	Derived neutrophil-to-lymphocyte ratio	N/A
	Platelet-to-lymphocyte ratio	N/A

^a^BCG: bacillus Calmette-Guérin.

^b^TUR: transurethral resection of a bladder tumor.

^c^Re-TUR: second-look transurethral resection of a bladder tumor.

^d^N/A: not applicable.

We additionally extracted medication information from the EHR for drugs that potentially interact with BCG, such as epirubicin (Table 1). Several medications that are used to prevent or manage BCG-associated adverse effects were extracted from medication administration and prescription data, including tuberculostatic agents, spasmolytics or anticholinergics, antiphlogistics, local topical steroids, cranberry supplements, and nonsteroidal anti-inflammatory drugs (Table 1) [19]. Patients who received BCG did not have documented use of cranberry supplements, and more contemporary medications such as pembrolizumab or atezolizumab were not available in the data during the time frame of this study [20]. The CUETO scoring model includes the variables of age, gender, number of tumors, tumor stage, grade, and presence of concomitant CIS. We investigated each variable included in the CUETO scoring model to determine if these risk factors were predictive of NMIBC recurrence in this cohort. We also investigated additional risk factors that have been previously demonstrated to predict NMIBC recurrence in the setting of BCG therapy such as perioperative chemotherapy agents, race, and diabetes (Multimedia Appendix 1) [21].

### Statistical Analysis

The 2016 American Urological Association (AUA) risk guidelines for recurrence were used to stratify each index TUR as low, medium, or high risk [22]. This stratification was used to describe cohort characteristics and BCG use. TURs identified as low risk were excluded from the remainder of the analysis because BCG was less likely to be clinically necessary or efficacious for these patients and thus rarely administered. Summary statistics were calculated using R version 3.5.2 (The R Foundation for Statistical Computing) and grouped by BCG use. The CUETO risk stratification tables for predicting recurrence were used as a multivariate measure of BCG efficacy because the CUETO scoring model was designed to consider BCG use as opposed to other NMIBC predictive models [9,23].

We used the Mann-Whitney U test—using the Wilcox test function in the stats package (version 1.8.12) in R—to compare patients who received BCG and were designated as intermediate to high risk for recurrence and BCG failure. Variables with a Mann-Whitney U value of <0.1 were considered candidates for multivariate logistic regression. Various combinations of multivariate logistic regression were tested using the generalized linear model function in the stats package in R, and the one with the highest area under the receiver operating characteristic curve was selected.

## Results

**Figure 1:**
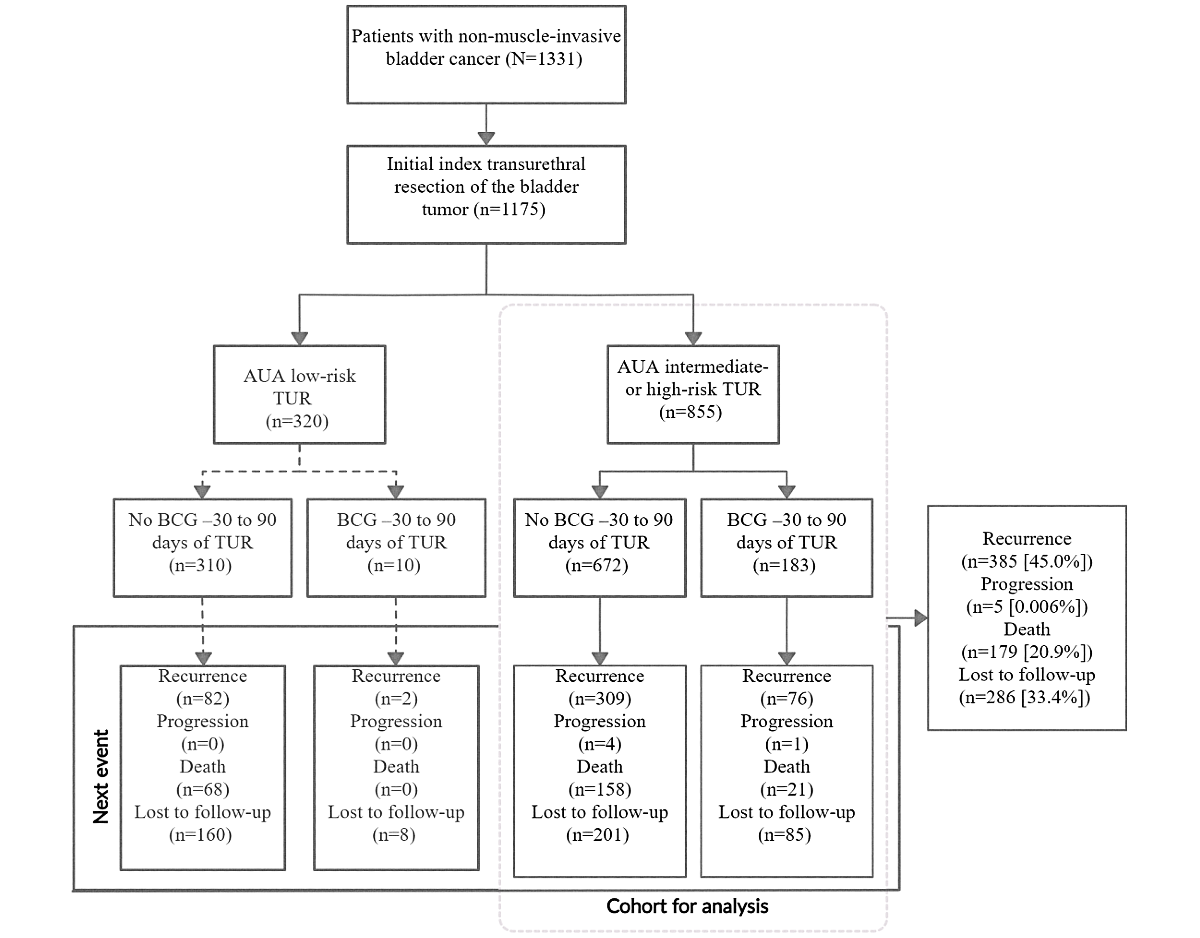
Patient flowchart. AUA: American Urological Association; BCG: bacillus Calmette-Guérin; TUR: transurethral resection of a bladder tumor.

Of the 1331 patients, 855 (64.24%) were intermediate to high risk according to the 2016 AUA guidelines, among whom 183 (21.4%) received an induction course of BCG (Figure 1; Table 2). Of the patients classified as intermediate to high risk who lost to follow-up (Figure 1), only 38 had a last check-in within 180 days of the index TUR (13 lost in less than 30 days; 8 lost between day 31 and day 60; 4 lost between day 61 and day 90; 6 lost between day 91 and day 120; and 7 lost between day 121 and day 180). In this cohort of 1331 patients with NMIBC, 105 (7.8%) progressed; however, all but the 5 included in Figure 1 followed at least one NMIBC recurrence event. The mean and median dates of BCG administration were 88 and 89 days, respectively, after the index TUR. White blood cell, monocyte and neutrophil counts as well as neutrophil-to-lymphocyte ratio measured between 1 and 180 days after BCG instillation were predictive of cancer recurrence in patients classified as intermediate and high risk who received BCG following an initial TUR (Table 3; Multimedia Appendix 1).

**Table 2:** Next event and cohort characteristics at the initial transurethral resection of the bladder tumor by bacillus Calmette-Guérin status.

Characteristic	Received BCG^a^ (n=183), n (%)	No BCG (n=672), n (%)	*P* value
Sex (male)	143 (78.1)	507 (75.5)	.45
White	175 (95.6)	652 (97)	.35
African American	6 (3.3)	14 (2.1)	.34
Hispanic ethnicity	3 (1.6)	9 (1.3)	.73
Other ethnicity	2 (1.1)	6 (0.9)	.68
Stage Ta	81 (44.3)	433 (64.4)	<.001
Stage T1	104 (56.8)	241 (35.7)	<.001
Low grade	34 (18.6)	320 (47.6)	<.001
High grade	149 (81.4)	354 (52.7)	<.001
Carcinoma in situ	99 (54.1)	99 (14.7)	<.001
Re-TUR^b^	32 (17.5)	63 (9.4)	.002
Mitomycin^c^	33 (18)	44 (6.5)	<.001
Cisplatin^c^	1 (0.5)	2 (0.3)	.51
Gemcitabine^c^	0 (0)	2 (0.3)	.99
Recurrence	76 (41.5)	309 (45.9)	.28
Progression	1 (0.5)	4 (0.6)	.99
Death	21 (11.5)	158 (23.5)	<.001

^a^BCG: bacillus Calmette-Guérin.

^b^Re-TUR: second-look transurethral resection of a bladder tumor.

^c^Chemotherapy agent used –30 to 90 days of initial index transurethral resection of the bladder tumor. There were no records of the use of lenalidomide, thiotepa, valrubicin, atezolizumab, and pembrolizumab.

**Table 3:** Club Urológico Español de Tratamiento Oncológico scoring model or statistically significant clinicopathological criteria in univariate analysis among patients with intermediate- or high-risk non–muscle-invasive bladder cancer who received bacillus Calmette-Guérin (N=183) +1 to 180 days after induction.

Clinicopathological criterion	Missing^a^, n (%)	Recurrence (Mann-Whitney U test)
Neutrophil count	73 (60.1)	0.009
Derived neutrophil-to-lymphocyte ratio	73 (60.1)	0.03
CUETO^b^, continuous	182 (99.5)	0.10
CUETO, categorical^c^	182 (99.5)	0.07
CUETO, gender	182 (99.5)	0.61
CUETO, number of tumors	182 (99.5)	0.05
CUETO, CIS^d^	182 (99.5)	0.33
CUETO, high-grade tumor	182 (99.5)	0.34
CUETO, age	183 (0)	0.75

^a^We have complete data for CUETO and age (N=183), and 0% of the data are missing. For other CUETO, we have data for 182 patients, and the data are 99.5% complete. We have 60.1% of data (from 73 patients) for neutrophil count within that time span, with 39.9% not having a recorded lab test for this in the timespan.

^b^CUETO: Club Urológico Español de Tratamiento Oncológico.

^c^CUETO, categorical: ≤4, 0 points; 5 or 6, 1 point; 7-9, 2 points; and ≥10, 3 points.

^d^CIS: carcinoma in situ.

**Figure 2:**
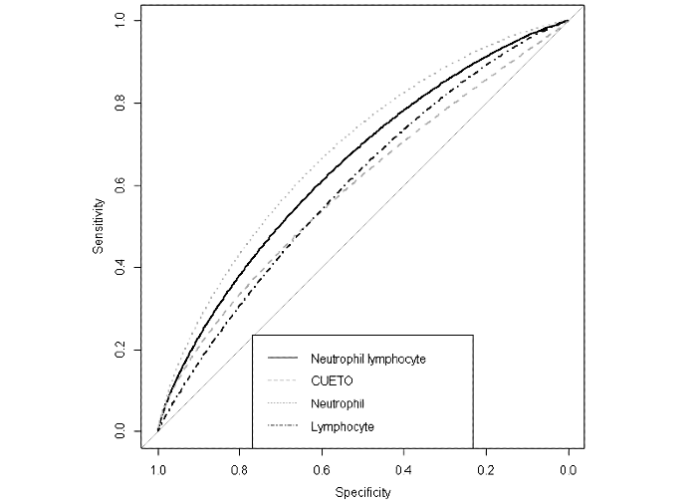
Area under the receiver operating characteristic curve for recurrence prediction. Time span: 1-180 days after bacillus Calmette-Guérin induction. CUETO: Club Urológico Español de Tratamiento Oncológico; Neutrophil: neutrophil count (differential); Lymphocyte: lymphocyte count (differential); Neutrophil Lymphocyte: derived neutrophil-to-lymphocyte ratio.

A univariate model with only a neutrophil-to-lymphocyte ratio of +1 to 180 days after BCG induction had an area under the receiver operating characteristic curve of 64.55% (Figure 2). Neither the CUETO scoring model nor any of its elements were found to be significant in univariate analysis in this cohort (Table 3) or when used as components in multivariate analysis with the neutrophil-to-lymphocyte ratio of +1 to 180 days after BCG induction (Table 4).

**Table 4:** Coefficients from multivariate regression with Club Urológico Español de Tratamiento Oncológico scoring model elements.

Coefficients	Estimate	SE	*t* test (*df*)	*P* value
(Intercept)	0.64586	0.18476	3.496 (66)	<.001
Gender	0.02581	0.04944	0.522 (175)	.60
Number of tumors	0.0927	0.08056	1.151 (175)	.25
Carcinoma in situ	–0.02382	0.07126	–0.334 (175)	.74
High-grade tumor	–0.07384	0.05686	–1.299 (175)	.20
Age	–0.12589	0.07109	–1.771 (175)	.08
Neutrophil-to-lymphocyte ratio	0.03017	0.01492	2.022 (66)	.047

## Discussion

### Principal Findings

Our main finding was that in patients with intermediate- or high-risk NMIBC who received BCG, the neutrophil-to-lymphocyte ratio measured between +1 and 180 days after BCG instillation was predictive of subsequent BCG failure. Early detection of BCG failure could prevent metastatic NMIBC progression by intervening earlier in tumor development. In addition, in the era of BCG shortages around the country, if patients elect to switch to BCG-salvage regimes, early detection of BCG failure would also preserve BCG supply for the patients who truly need it. In this study, we evaluated whether common clinicopathological variables might be predictive of BCG failure in patients with elevated risk. BCG failure is broadly categorized as refractory, relapsing (ie, recurrence), unresponsive, and intolerant cases [24-26]. However, we did not have information on BCG intolerance and, therefore, did not include this information in our analysis. These results suggest that white blood cell, monocyte, and neutrophil counts as well as the neutrophil-to-lymphocyte ratio measured between 1 and 180 days after BCG instillation were as predictive of cancer recurrence in patients classified as intermediate and high risk who received BCG following an initial TUR while adjusting for the CUETO score. This suggests that monitoring blood panels is useful in the first 6 months after BCG instillation to evaluate whether BCG failure is likely to occur. In addition, neutrophil count alone, when measured within 180 days of BCG instillation, provides predictive performance equivalent to that of monocyte count, neutrophil-to-lymphocyte ratio, or combinations of these variables.

For patients who undergo BCG therapy, the CUETO risk model was designed to predict the probability of cancer recurrence and progression. However, in this cohort, the CUETO score was not a statistically significant differentiator for predicting recurrence after patients classified as low risk (AUA risk guidelines) were excluded from the cohort. This could be due to the poor generalizability of the CUETO scoring model. When external data were previously applied to these scores, the CUETO scoring model overestimated disease recurrence and demonstrated a poor ability to predict recurrence [10-12]. Perhaps, most importantly, these results show that even when the CUETO elements were included in the multivariate analysis, the neutrophil-to-lymphocyte ratio remains a robust predictor. This provides preliminary evidence that in a diverse cohort of patients with NMIBC treated with BCG, the neutrophil-to-lymphocyte ratio could help guide treatment decision making, particularly for NMIBC surveillance. If patients with elevated risk demonstrate a neutrophil-to-lymphocyte ratio indicative of BCG failure, they might benefit from more frequent cystoscopy, enhanced cystoscopy techniques, or additional imaging procedures. This is similar to current AUA guideline recommendations for patients with high-risk disease who show positive cytology during surveillance [22]. Additional prospective studies are warranted to determine the predictive power of the neutrophil-to-lymphocyte ratio in a large randomized sample of patients with NMIBC.

The clinicopathological criteria—neutrophils and lymphocytes—are consistent with those in the study by Vartolomei et al [15]; however, the time frame of measurement differs, which deserves further investigation. As BCG is known to be immunostimulatory, markers of net functional immunity, such as the neutrophil-to-lymphocyte ratio, are affected by its use. Although these markers of net functional immunity are nonmodifiable, they can be useful clinically to predict the failure of the BCG treatment strategy by informing the clinician that recurrence is likely, and another clinical intervention is required.

A second course of BCG induction may be reasonable for refractory or relapsing cases because approximately 25%-50% of these patients will respond to this subsequent induction [24-27]. We concur with Zamboni et al [10] that the completeness of BCG schedules may be useful to include in future models for optimizing BCG use across the entire course, rather than just the induction. Future studies with sufficient power to capture data for all subclassifications of BCG failure (particularly progression) or following a second course of BCG induction may provide additional insights into predicting BCG efficacy.

Our data sets only contain data for patients before the halt in the production of the Connaught BCG strain by Sanofi Pasteur, thus precluding any impact analysis, although we expect that these effects are likely more recent than the 2017 stoppage, and we are unaware of the date corresponding to when stockpiles of that strain became unavailable [3,28]. Several clinical recommendations on how to manage patients with NMIBC when BCG supplies are low can be found in the clinical literature [1,2]. Patients at increased risk for BCG failure could undergo additional surveillance, receive maintenance intravesical chemotherapy in addition to induction BCG, or become candidates for timely cystectomy. Given the poor generalizability of the CUETO scoring model in the literature [10-12], external validation of these results is warranted before recommending additional expenditure on complete blood panels that are requested outside of the current standard of care.

Continuous monitoring of a patient’s clinicopathological criteria for the duration of NMIBC treatment to predict future recurrence events is a unique aspect of this study. Another unique aspect was to account for differences in markers of net functional immunity (eg, neutrophil count and lymphocyte count) over time, effectively evaluating whether the changes are predictive. Although the changes in neutrophil count were significant in univariate analysis (Table 3), the value of the neutrophil count at +1 to 180 days after BCG induction was sufficient to account for this change in the multivariate analysis. The temporality of when things are measured with respect to an event (eg, BCG induction) is important when considering risk factors because the time period from –90 to 0 days before BCG induction and >180 days after BCG induction were not found to be predictive of recurrence when considering these clinicopathological criteria (Multimedia Appendix 1).

### Limitations

Prior research by Tazeh et al [29] suggests that race-specific differences should be considered when interpreting the neutrophil-to-lymphocyte ratio at the time of TUR. However, the study population lacks sufficient diversity to thoroughly investigate this finding [29]. As only data from one health system were included, these statistics may not generalize elsewhere without adaptation to the local institution’s EHR. The disadvantage of a retrospective cohort design is that data may be incomplete or inadequately captured in the available medical record data [30]. For example, BCG dose information was scarce in these data because only a portion of the records was associated with pharmaceutical records, and the proportion of patients with a known lower dose (eg, a one-third dose) was too small for a subgroup analysis to be performed. Outside of a clinical trial, it is unlikely that missing data would be collected more frequently in future clinical workflows without changes to meaningful use requirements or documentation guidelines; thus, the statistics are more robust when applied to typical clinical environments. The consumption of steroids or the presence of an infection or thromboembolism, any of which may affect the neutrophil-to-lymphocyte ratio, were not considered in this study. Smoking status, which has been shown to predict recurrence [31,32], was also not considered because our retrospective data lacked completeness and granularity of smoking status.

### Conclusions

In patients with intermediate- or high-risk NMIBC who received BCG, the neutrophil-to-lymphocyte ratio measured between +1 and 180 days after BCG instillation was predictive of subsequent BCG failure. In conjunction with existing risk stratification scores such as the CUETO score, the neutrophil-to-lymphocyte ratio could be used to predict BCG failure. Patients at increased risk for BCG failure could undergo additional surveillance, receiving maintenance intravesical chemotherapy instead of BCG, thereby preserving limited BCG supplies, or be considered for timely cystectomy. Additional retrospective and prospective studies are needed to validate these findings.

## Appendix

Multimedia Appendix 1 Additional statistical analysis at different time points.

## Abbreviations

AUA: American Urological Association
BCG: bacillus Calmette-Guérin
CIS: carcinoma in situ
CUETO: Club Urológico Español de Tratamiento Oncológico
EHR: electronic health record
IRB: institutional review board
NMIBC: non–muscle-invasive bladder cancer
TUR: transurethral resection of a bladder tumor
