# Supplementary material for: Clinicopathological Criteria Predictive of Recurrence Following Bacillus Calmette-Guérin Therapy Initiation in Non–Muscle-Invasive Bladder Cancer: Retrospective Cohort Study
Source: JMIR Cancer. 2021 Jun 22;7(2):e25800. doi: 10.2196/25800 (PMC8277393; doi:10.2196/25800)
Supplement: Multimedia Appendix 1 [file cancer_v7i2e25800_app1.docx]

**Multimedia Appendix 1:** Additional statistical analysis at different timepoints

Methods: Several clinical characteristics associated with the efficacy of BCG treatment were extracted from the EHR, including white blood cell, lymphocyte, neutrophil, monocyte, and platelet counts and levels of creatinine and hemoglobin A1c (Table A1). Derived neutrophil-to-lymphocyte ratio and platelet-to-lymphocyte ratio were computed based on EHR data. A delta in laboratory values over three different time periods was calculated in order to further evaluate if a change in patients’ clinical characteristics between the time periods demonstrated any clinical relevance to predicting recurrence.

Multimedia Appendix Table 1. Clinicopathological criteria or medication definitions

| Clinicopathological criteria, comorbidity, or medication | Type | Description |
| --- | --- | --- |
| BCG instillation | Binary | First instillation of BCG documented 0 to 90 days post-TUR |
| Lymphocyte count (normal: 20-40% differential) | Numeric | 1. Most recent [lab test] occurring -90 to 0 days pre-induction BCG (index to median day of BCG administration if no BCG) 2. Most recent [lab test] occurring +1 to 180 days post-induction BCG commuted 14 days prior to the next event (i.e., TUR but not Re-TUR, recurrence, progression, death) 3. Most recent [lab test] beyond 180 days of induction BCG commuted 14 days prior to next event (i.e., TUR but not Re-TUR, recurrence, progression, death) 4. Difference between the most recent [lab test] occurring beyond 180 days post-induction BCG commuted 14 days prior to next event and the most recent [lab test] occurring -90 to 0 days pre-induction BCG (index to median day of BCG administration if no BCG) 5. Difference between the most recent [lab test] occurring +1 to 180 days post-induction BCG commuted 14 days prior to next event and the most recent [lab test] occurring -90 to 0 days pre-induction BCG (index to median day of BCG administration if no BCG) 6. Difference between the most recent [lab test] occurring beyond 180 days post-induction BCG commuted 14 days prior to next event and the most recent [lab test] occurring +1 to 180 days pre-induction BCG (index to median day of BCG administration if no BCG) |
| Neutrophil count (normal: 55-70% differential) |  |  |
| Monocyte count (normal: 2-8% differential) |  |  |
| Platelet count (K/μL) |  |  |
| White blood cell count (K/μL) |  |  |
| Creatinine level (mg/dL) |  |  |
| Hemoglobin A1c (mmol/mol) |  |  |
| Derived neutrophil-to-lymphocyte ratio | Computed percentage (%) | 1. Most recent [lab test] occurring -90 to 0 days pre-induction BCG (index to median day of BCG administration if no BCG) 2. Most recent [lab test] occurring +1 to 180 days post- induction BCG commuted 14 days prior to the next event (i.e., TUR but not Re-TUR, recurrence, progression, death) 3. Most recent [lab test] beyond 180 days of induction BCG commuted 14 days prior to next event (i.e., TUR but not Re-TUR, recurrence, progression, death) 4. Difference between the most recent [lab test] occurring beyond 180 days post-induction BCG commuted 14 days prior to next event and the most recent [lab test] occurring -90 to 0 days pre-induction BCG (index to median day of BCG administration if no BCG) 5. Difference between the most recent [lab test] occurring +1 to 180 days post-induction BCG commuted 14 days prior to next event and the most recent [lab test] occurring -90 to 0 days pre-induction BCG (index to median day of BCG administration if no BCG) 6. Difference between the most recent [lab test] occurring beyond 180 days post-induction BCG commuted 14 days prior to next event and the most recent [lab test] occurring +1 to 180 days pre-induction BCG (index to median day of BCG administration if no BCG) |
| Platelet-to-lymphocyte ratio |  |  |
| Epirubicin | Binary | Utilization documented 0 to 90 days post TUR |
| Tuberculostatic agents | Binary | Utilization of Isoniazid isonicotinylhydrazide (INH), rifampicin, rifambutin, fluoroquinolones (ofloxacin, ciprofloxacin, levofloxacin, moxifloxacin), ethambutol, clarithromycin, aminoglycosides (gentamicin, amikacin, tobramycin, kanamycin, neomycin), or doxycycline documented -30 to 90 days of induction BCG (index to median day of BCG administration if no BCG) |
| Spasmolytics/anticholinergics | Binary | Utilization of spasmolytics: oxybutynin documented -30 to 90 days of induction BCG (index to median day of BCG administration if no BCG) |
| Antiphlogistics | Binary | Utilization of antiphlogistics: fluticasone documented -30 to 90 days of induction BCG (index to median day of BCG administration if no BCG) |
| Topical steroids | Binary | Utilization of local topical steroids: betamethasone, clobetasol, diflorasone, fluocinoide, halobetasol, amcinonide, desoximetasone, propionate, triamcinolone, fluocinolone, hydrocortisone, desonide, alclometasone, mometasone documented -30 to 90 days of induction BCG (index to median day of BCG administration if no BCG) |
| Nonsteroidal anti-inflammatory drugs | Binary | Utilization of nonsteroidal anti-inflammatory drugs:  Aspirin, ibuprofen, naproxen, nabumetone, celecoxib, diclofenac, etodolac, indomethacin, ketoprofen, ketorolac, piroxicam documented -30 to 90 days of induction BCG (index to median day of BCG administration if no BCG) |
| Mitomycin | Binary | Utilization documented 0 to 90 days post TUR |
| Gemcitabine | Binary | Utilization documented 0 to 90 days post TUR |
| Cisplatin | Binary | Utilization documented 0 to 90 days post TUR |
| Fluorouracil | Binary | Utilization documented 0 to 90 days post TUR |
| Doxorubicin | Binary | Utilization documented 0 to 90 days post TUR |
| Carboplatin | Binary | Utilization documented 0 to 90 days post TUR |
| Docetaxel | Binary | Utilization documented 0 to 90 days post TUR |
| Type1diabetes | Binary | Comorbidity documented <= 365 days post TUR |
| Type2diabetes | Binary | Comorbidity documented <= 365 days post TUR |

Multimedia Appendix Table 2. Additional summary statistics for clinicopathological criteria or medication for intermediate- to high-risk patients who received Bacillus Calmette-Guérin induction

| N=183 | Timespan (days post-BCG induction) | n (% missing) | Mean | Standard deviation | Range | Skew | Kurtosis | Standard error of mean | Recurrence  Mann-Whitney U |
| --- | --- | --- | --- | --- | --- | --- | --- | --- | --- |
| Lymphocyte count | -90 to 0 | 97 (47.0) | 24.1 | 8.58 | [8,45] | 0.27 | -0.36 | 0.87 | .77 |
| Lymphocyte count | +1 to 180 | 73 (60.1) | 21.98 | 9.9 | [0.8,51] | 0.55 | 0.45 | 1.16 | .09 |
| Lymphocyte count | >+180 | 84 (54.1) | 22.04 | 11.98 | [1,83] | 2.0 | 7.4 | 1.31 | .43 |
| Lymphocyte count | (+1 to 180)-  (-90 to 0) | 49 (73.2) | 0.41 | 7.61 | [-19,25] | 0.42 | 2.08 | 1.09 | .42 |
| Lymphocyte count | (>+180)-  (-90 to 0) | 50 (72.7) | -1.66 | 12.46 | [-35,42] | 0.45 | 2.23 | 1.76 | .99 |
| Lymphocyte count | (>+180)-(+1 to 180) | 39 (78.7) | -0.71 | 8.24 | [-16,38] | 2.37 | 10.1 | 1.32 | .36 |
| Neutrophil count | -90 to 0 | 97 (47.0) | 63.98 | 10.21 | [43,91] | 0.22 | -0.25 | 1.04 | .55 |
| Neutrophil count | +1 to 180 | 73 (60.1) | 63.94 | 13.2 | [2.9,91] | -1.23 | 4.72 | 1.54 | .009 |
| Neutrophil count | >+180 | 84 (54.1) | 64.51 | 14.84 | [4,94] | -1.46 | 3.82 | 1.62 | .49 |
| Neutrophil count | (+1 to 180)-  (-90 to 0) | 49 (73.2) | -1.18 | 9.84 | [-25,26] | 0.2 | 1.28 | 1.41 | .13 |
| Neutrophil count | (>+180)-  (-90 to 0) | 50 (72.7) | 1.82 | 14.48 | [-32,41] | 0.08 | 0.69 | 2.05 | .92 |
| Neutrophil count | (>+180)-(+1 to 180) | 39 (78.7) | 2.28 | 10.61 | [-34,33] | -0.31 | 3.11 | 1.7 | .023 |
| Monocyte count | -90 to 0 | 97 (47.0) | 8.88 | 3.17 | [1,26] | 1.66 | 7.89 | 0.32 | .84 |
| Monocyte count | +1 to 180 | 73 (60.1) | 9.71 | 4.12 | [0.7,34] | 2.84 | 14.69 | 0.48 | .13 |
| Monocyte count | >+180 | 84 (54.1) | 9.04 | 3.1 | [1.1,18] | 0.13 | 0.43 | 0.34 | .69 |
| Monocyte count | (+1 to 180)-  (-90 to 0) | 49 (73.2) | 0.8 | 4.56 | [-8,25] | 2.78 | 13.74 | 0.65 | .05 |
| Monocyte count | (>+180)-  (-90 to 0) | 50 (72.7) | -0.18 | 4.41 | [-16,8] | -1.3 | 2.88 | 0.62 | .77 |
| Monocyte count | (>+180)-(+1 to 180) | 39 (78.7) | -1.32 | 5.05 | [-25,10] | -2.38 | 10.34 | 0.81 | .13 |
| Platelet count | -90 to 0 | 85 (53.6) | 223.84 | 60.14 | [53,383] | 0.06 | 0.37 | 6.52 | .35 |
| Platelet count | +1 to 180 | 47 (74.3) | 227.7 | 70.15 | [45,428] | 0.35 | 0.62 | 10.23 | .17 |
| Platelet count | >+180 | 39 (78.7) | 212.67 | 81.71 | [4.3,420] | 0.14 | 0.46 | 13.08 | .25 |
| Platelet count | (+1 to 180)- (-90 to 0) | 37 (79.8) | 3.76 | 34.48 | [-84,103] | 0.07 | 0.94 | 5.67 | .53 |
| Platelet count | (>+180)-  (-90 to 0) | 31 (83.1) | -6.68 | 42.69 | [-102,126] | 0.67 | 1.49 | 7.67 | .62 |
| Platelet count | (>+180)-(+1 to 180) | 21 (88.5) | 1.67 | 45.05 | [-68,108] | 0.86 | 0.19 | 9.83 | .90 |
| White blood cell Count | -90 to 0 | 156 (14.8) | 7.48 | 2.49 | [3.3,21.4] | 1.73 | 5.97 | 0.2 | .89 |
| White blood cell count | +1 to 180 | 96 (47.5) | 7.65 | 2.53 | [2.5,14.3] | 0.82 | 0.14 | 0.26 | .06 |
| White blood cell Count | >+180 | 94 (48.6) | 7.58 | 3.07 | [1,26] | 2.5 | 12.12 | 0.32 | .70 |
| White blood cell count | (+1 to 180)-(-90 to 0) | 83 (54.6) | 0.08 | 2.12 | [-8.6,5.5] | -0.18 | 3.19 | 0.23 | .006 |
| White blood cell count | (>+180)-  (-90 to 0) | 81 (55.7) | 0.4 | 3.1 | [-6.6,20.5] | 3.22 | 19.78 | 0.34 | .18 |
| White blood cell count | (>+180)-(+1 to 180) | 55 (69.9) | 0.51 | 1.67 | [-2.6,4.4] | 0.68 | -0.16 | 0.23 | .10 |
| Creatinine level | -90 to 0 | 164 (10.4) | 1.17 | 0.46 | [0.58,5] | 4.16 | 28.4 | 0.04 | .36 |
| Creatinine level | +1 to 180 | 113 (38.3) | 4.46 | 35.08 | [0.5,374] | 10.35 | 106.03 | 3.3 | .75 |
| Creatinine level | >+180 | 108 (41.0) | 2.64 | 14.99 | [0.46,156.8] | 10.08 | 100.66 | 1.44 | .20 |
| Creatinine level | (+1 to 180)-(-90 to 0) | 103 (43.7) | 3.58 | 36.36 | [-0.6,369] | 9.85 | 96.05 | 3.58 | .99 |
| Creatinine level | (>+180)-  (-90 to 0) | 97 (47.0) | 1.63 | 15.82 | [-0.7,155.8] | 9.54 | 89.98 | 1.61 | .50 |
| Creatinine level | (>+180)-(+1 to 180) | 77 (57.9) | -2.72 | 45.69 | [-367.4,155.6] | -6.06 | 51.59 | 5.21 | .98 |
| Hemoglobin A1c | -90 to 0 | 25 (86.3) | 6.88 | 1.11 | [5.2,9.7] | 0.88 | -0.03 | 0.22 | .39 |
| Hemoglobin A1c | +1 to 180 | 22 (88.0) | 7.32 | 1.15 | [5.8,9.9] | 0.82 | -0.46 | 0.24 | .18 |
| Hemoglobin A1c | >+180 | 37 (79.8) | 6.37 | 1.17 | [4,10.3] | 1.13 | 2.42 | 0.19 | .37 |
| Hemoglobin A1c | (+1 to 180)-(-90 to 0) | 13 (92.9) | 0.3 | 0.74 | [-1.3,1.7] | -0.18 | -0.11 | 0.21 | .69 |
| Hemoglobin A1c | (>+180)-  (-90 to 0) | 13 (92.9) | -0.02 | 0.78 | [-1.1,1.6] | 0.68 | -0.4 | 0.22 | NA |
| Hemoglobin A1c | (>+180)-(+1 to 180) | 16 (91.3) | -0.02 | 0.98 | [-1.8,2.1] | 0.39 | -0.26 | 0.24 | NA |
| Derived neutrophil-to-lymphocyte ratio | -90 to 0 | 97 (47.0) | 3.29 | 2.09 | [0.96,11.38] | 1.78 | 3.17 | 0.21 | .71 |
| Derived neutrophil-to-lymphocyte ratio | +1 to 180 | 73 (60.1) | 4.04 | 3.89 | [0.71,30.33] | 4.44 | 26.04 | 0.46 | .03 |
| Derived neutrophil-to-lymphocyte ratio | >+180 | 84 (54.1) | 5.01 | 10.22 | [0.17,94] | 7.86 | 65.41 | 1.12 | .47 |
| Derived neutrophil-to-lymphocyte ratio | (+1 to 180)  -(-90 to 0) | 49 (73.2) | 37.3 | 16.43 | [-8.92,66.89] | -0.62 | 0.36 | 2.35 | .08 |
| Derived neutrophil-to-lymphocyte ratio | (>+180)-  (-90 to 0) | 50 (72.7) | 35.57 | 16.12 | [-27.53,62.62] | -1.59 | 3.55 | 2.28 | .27 |
| Derived neutrophil-to-lymphocyte ratio | (>+180)-(+1 to 180) | 39 (78.7) | 35.06 | 20.01 | [-31.58,63.69] | -1.25 | 1.8 | 3.2 | .58 |
| Platelet-to-lymphocyte ratio | -90 to 0 | 33 (82.0) | 10.25 | 6.88 | [2.07,32.5] | 1.76 | 2.72 | 1.2 | .24 |
| Platelet-to-lymphocyte ratio | +1 to 180 | 26 (85.8) | 18.89 | 30.96 | [3.21,166.25] | 4.13 | 16.76 | 6.07 | .37 |
| Platelet-to-lymphocyte ratio | >+180 | 31 (83.1) | 12.2 | 7.57 | [0.19,36.57] | 1.43 | 2.3 | 1.36 | .81 |
| Platelet-to-lymphocyte ratio | (+1 to 180)  -(-90 to 0) | 12 (93.4) | 178.17 | 74.86 | [25.21,311.26] | -0.15 | -0.47 | 21.61 | .14 |
| Platelet-to-lymphocyte ratio | (>+180)-  (-90 to 0) | 12 (93.4) | 177.89 | 51.49 | [102.1,256.14] | -0.19 | -1.44 | 14.87 | .37 |
| Platelet-to-lymphocyte ratio | (>+180)-(+1 to 180) | 7 (96.2) | 157.32 | 66.52 | [58.58,252.5] | -0.07 | -1.59 | 25.14 | .19 |
| Epirubicin | 0 to 90 post TUR | 183 (0) | 0.03 | 0.18 | {0,1} | 5.2 | 25.22 | 0.01 | .68 |
| Tuberculostatic agents | -30 to 90 | 183 (0) | 0.95 | 0.22 | {0,1} | -4.14 | 15.18 | 0.02 | .61 |
| Spasmolytics | -30 to 90 | 183 (0) | 0.1 | 0.31 | {0,1} | 2.58 | 4.66 | 0.02 | .30 |
| Topical steroids | -30 to 90 | 183 (0) | 0.29 | 0.45 | {0,1} | 0.92 | -1.16 | 0.03 | .75 |
| Nonsteroidal anti-inflammatory | -30 to 90 | 183 (0) | 0.73 | 0.45 | {0,1} | -1.01 | -0.99 | 0.03 | .21 |
| CUETO (continuous) | +1 to 180 | 182 (99.5) | 4.76 | 1.99 | [0,9] | 0 | -0.15 | 0.15 | .10 |
| CUETO (categorical) | +1 to 180 | 182 (99.5) | 0.77 | 0.79 | {0,1,2} | 0.41 | -1.28 | 0.06 | .07 |
| CUETO_GENDER | +1 to 180 | 182 (99.5) | 0.76 | 1.31 | {M,F} | 1.13 | -0.72 | 0.04 | .61 |
| CUETO_NUMTUMORS | +1 to 180 | 182 (99.5) | 0.14 | 0.51 | {0,1} | 3.35 | 9.22 | 0.02 | .05 |
| CUETO_CIS | +1 to 180 | 182 (99.5) | 0.11 | 0.45 | {0,1} | 3.94 | 13.53 | 0.01 | .33 |
| CUETO_HIGHGRADE | +1 to 180 | 182 (99.5) | 1.28 | 1.48 | {0,1,2} | 0.29 | -1.92 | 0.04 | .34 |
| CUETO_AGE | +1 to 180 | 183 (0) | 1.38 | 0.77 | {0,1} | -0.78 | -0.9 | 0.02 | .75 |
| Mitomycin | 0 to 90 post TUR | 183 (0) | 0.07 | 0.26 | {0,1} | 3.35 | 9.24 | 0.01 | .22 |
| Gemcitabine | 0 to 90 post TUR | 183 (0) | 0 | 0.03 | {0,1} | 33.81 | 1142.01 | 0 |  |
| Cisplatin | 0 to 90 post TUR | 183 (0) | 0 | 0.05 | {0,1} | 19.47 | 377.34 | 0 | .40 |
| Fluorouracil | 0 to 90 post TUR | 183 (0) | 0 | 0.03 | {0,1} | 33.81 | 1142.01 | 0 | .24 |
| Doxorubicin | 0 to 90 post TUR | 183 (0) | 0.03 | 0.17 | {0,1} | 5.37 | 26.9 | 0.01 | .70 |
| Carboplatin | 0 to 90 post TUR | 183 (0) | 0 | 0.05 | {0,1} | 19.47 | 377.34 | 0 |  |
| Docetaxel | 0 to 90 post TUR | 183 (0) | 0 | 0.03 | {0,1} | 33.81 | 1142.01 | 0 |  |
| Type1diabetes | <= TUR + 365 | 183 (0) | 0.03 | 0.16 | {0,1} | 5.94 | 33.26 | 0 | .75 |
| Type2diabetes | <= TUR + 365 | 183 (0) | 0.19 | 0.4 | {0,1} | 1.55 | 0.39 | 0.01 | .70 |

Multimedia Appendix Table 3. Next event and cohort characteristics at the initial transurethral resection of the bladder tumor

|  | Intermediate-risk AUA | | High-risk AUA | |
| --- | --- | --- | --- | --- |
|  | Yes BCG  (n = 51) | No BCG  (n = 401) | Yes BCG  (n = 132) | No BCG  (n = 271) |
| **Characteristic** |  |  |  |  |
| Mean age [range] | 66 [36-88] | 71 [22-97] | 70 [43-98] | 74 [30-96] |
| Sex = male | 36 (70.6 %) | 300 (74.8 %) | 107 (81.1%) | 207 (76.4%) |
| Caucasian | 48 (94.1%) | 393 (98.0%) | 127 (96.2%) | 259 (95.6%) |
| African American | 3 (5.8%) | 6 (1.5%) | 3 (2.3%) | 8 (2.9%) |
| Hispanic ethnicity | 0 (0%) | 5 (1.2%) | 3 (2.3%) | 4 (1.4%) |
| Other ethnicity | 0 (0%) | 2 (0.5%) | 2 (1.5%) | 4 (1.4%) |
| Stage Ta | 44 (86.3%) | 351 (87.5%) | 37 (28.0%) | 82 (30.3%) |
| Stage T1 | 8 (15.7%) | 50 (12.5%) | 96 (72.7%) | 191 (70.5%) |
| Low grade | 30 (58.8%) | 308 (76.8%) | 4 (3.0%) | 12 (4.4%) |
| High grade | 21 (41.2%) | 94 (23.4%) | 128 (97.0%) | 260 (95.9%) |
| Carcinoma in situ | 0^a^ | 0^a^ | 99 (75.0%) | 99 (36.5%) |
| Re-TUR following Index TUR | 5 (9.8%) | 19 (4.7%) | 27 (20.4%) | 44 (16.2%) |
| Mitomycin^b^ | 11 (21.6%) | 24 (5.6%) | 22 (16.7%) | 20 (8.1%) |
| Cisplatin^b^ | 1 (2.0%) | 0 (0%) | 0 (0%) | 2 (0.7%) |
| Gemcitabine^b^ | 0 (0%) | 1 (0.2%) | 0 (0%) | 1 (0.3%) |
| **Next Event** | | | | |
| Recurrence | 15 (29.4%) | 195 (48.6%) | 61 (46.2%) | 114 (42.1%) |
| Progression | 0 (0%) | 1 (0.2%) | 1 (0.8%) | 3 (1.1%) |
| Death | 8 (15.7%) | 79 (19.7%) | 13 (9.8%) | 79 (29.2%) |
| Lost to follow-up | 28 (54.9%) | 126 (31.4%) | 57 (43.2%) | 75 (27.7%) |

^a^Per American Urological Association (AUA) definition.

^b^Chemotherapy agent utilized -30 to 90 days of initial index transurethral resection of the bladder tumor (TUR). There were no records of utilization of lenalidomide, thiotepa, valrubicin atezolizumab, and pembrolizumab. Abbreviation: BCG = Bacillus Calmette-Guérin.

Multimedia Appendix Table 4. Next event and cohort characteristics at the initial transurethral resection of the bladder tumor by American Urological Association classification

| Characteristic | Intermediate-risk AUA^a^  (n = 452) | High-risk AUA^a^  (n = 403) | *P* | Test |
| --- | --- | --- | --- | --- |
| Sex = male | 336 (74.34%) | 314 (77.92%) | .22 | Chi-square |
| Caucasian | 441 (97.57%) | 386 (95.78%) | .14 | Chi-square |
| African American | 9 (1.99%) | 11 (2.73%) | .48 | Chi-square |
| Hispanic ethnicity | 5 (1.11%) | 7 (1.74%) | .56 | Fisher’s Exact |
| Other ethnicity | 2 (0.44%) | 6 (1.49%) | .16 | Fisher’s Exact |
| Stage Ta | 395 (87.39%) | 119 (29.53%) | <.001 | Chi-square |
| Stage T1 | 58 (12.83%) | 287 (71.22%) | <.001 | Chi-square |
| Low grade | 338 (74.78%) | 16 (3.97%) | <.001 | Chi-square |
| High grade | 115 (25.44%) | 388 (96.28%) | <.001 | Chi-square |
| Carcinoma in situ | 0^a^ | 198 (49.13%) | Not applicable | Not applicable |
| Re-TUR following Index TUR | 24 (5.31%) | 71 (17.62%) | <.001 | Chi-square |
| Mitomycin^b^ | 35 (7.74%) | 42 (10.42%) | .17 | Chi-square |
| Cisplatin^b^ | 1 (0.22%) | 2 (0.5%) | .60 | Fisher’s Exact |
| Gemcitabine^b^ | 1 (0.22%) | 1 (0.25%) | .99 | Fisher’s Exact |
| Recurrence | 210 (46.46%) | 175 (43.42%) | .37 | Chi-square |
| Progression | 1 (0.22%) | 4 (0.99%) | .60 | Fisher’s Exact |
| Death | 87 (19.25%) | 92 (22.83%) | .20 | Chi-square |
| Lost to follow-up | 154 (34.07%) | 132 (32.75%) | .68 | Chi-square |

^a^Per American Urological Association (AUA) definition.

^b^Chemotherapy agent utilized -30 to 90 days of initial index transurethral resection of the bladder tumor (TUR). There were no records of utilization of lenalidomide, thiotepa, valrubicin atezolizumab, and pembrolizumab.
